# Macrophage polarization in experimental and clinical choroidal neovascularization

**DOI:** 10.1038/srep30933

**Published:** 2016-08-04

**Authors:** Yu Yang, Fang Liu, Miao Tang, Miner Yuan, Andina Hu, Zongyi Zhan, Zijing Li, Jiaqing Li, Xiaoyan Ding, Lin Lu

**Affiliations:** 1 State Key Laboratory of Ophthalmology, Retina Division, Zhongshan Ophthalmic Center, Sun Yat-sen University, 510060, Guangzhou, Guangdong, China

## Abstract

Macrophages play an important role in the development of age-related macular degeneration (AMD). In this study, the spatial and temporal changes and the polarization of macrophages in murine laser-induced choroidal neovascularization (CNV) were investigated, and the polarized M1 and M2 biomarkers in the aqueous humors of neovascular AMD (nAMD) patients were studied. Macrophages, the main infiltrating inflammatory cells in CNV lesions, were evidenced by a significant increase in F4/80 mRNA expression and by the infiltration of F4/80+ cells in the lesions and the vicinity of laser-induced CNV. The mRNA expressions of M1-related markers were dramatically upregulated in the early stage, while the M2-related markers were slightly upregulated in the middle stage and sustained until the late stage. The results of immunostaining showed a similar early-but-transient M1 pattern and a delayed-but-sustained M2 pattern in laser-induced CNV. In addition, a higher M2/M1 ratio was found in both the murine models (*Arg-1/iNOS* and *CCL22/CXCL10*) and the aqueous humors of nAMD patients (*CCL22/CXCL10*) than in the controls. Our results suggested that the dynamic patterns of M1 and M2 were different in both the experimental and clinical CNV. The M2 macrophages were predominant and may play a more important role in the development of CNV.

Age-related macular degeneration (AMD) is the leading cause of irreversible central vision loss in the elderly population worldwide. Advanced AMD is generally classified into two categories: geographic atrophy (aAMD) or ‘dry’ AMD, and neovascular (nAMD) or ‘wet’ AMD. nAMD leads to irreversible vision loss in 90% of patients through neovascularization from the choroid, secondary subretinal hemorrhage, photoreceptor cell death, and ultimately, irreversible fibrovascular scarring. This process is characterized by the proliferation and/or infiltration of various types of cells, including retinal pigment epithelium (RPE) cells, glial cells, fibroblasts and macrophages, which interact with inflammatory cytokines and growth factors^1^. The initial evidence linking macrophages to AMD came from an analysis of mice deficient in macrophage chemokine signaling components (*ccl2*^−*/*−^ and *ccr2*^−*/*−^mice), which showed retinal defects similar to AMD with advanced age (2 years or older), including spontaneous choroidal neovascularization (CNV) and drusen formation^2^. Furthermore, abundant evidence from human and rodent studies has demonstrated that macrophages are the main infiltrating inflammatory cells in AMD lesions, particularly in the CNV membranes, indicating a prominent role for macrophages in AMD development^3^,^4^,^5^,^6^,^7^,^8^.

Macrophages have been proved to be able to be polarized, and have been characterized based on their functions as surface markers and their cytokine/chemokine profiles. Classically activated macrophages (M1) and alternatively activated macrophages (M2) are at the opposite ends of this spectrum. M1 macrophages, driven by Th1 cytokines, are generally proinflammatory and secrete M1 chemokines, such as CXCL10 and CXCL11 (chemotactic for activated T cells). In contrast, M2 macrophages, driven by Th2 cytokines, facilitate tissue repair and neovascularization, and secrete M2 chemokines, such as CCL2 and CCL22 (chemoattractants for Th2 cells that enhance fibrosis). However, due to their plasticity, macrophages with one phenotype can easily convert into another phenotype when placed in a new microenvironment. Therefore, identifying the temporal and spatial changes of macrophages during the early and late stages of nAMD can lead to a better understanding of its pathogenesis. However, to date, the phenotype, number, and distribution of macrophages in nAMD remains unknown. In this study, we investigated the spatial and temporal changes of macrophages and the polarized M1 and M2 markers in laser-induced nAMD-like CNV in mice, and in the aqueous humors of human nAMD patients.

## Results

### Mouse Model of Laser-induced CNV

Laser-induced CNV in mice is currently the most established and commonly utilized model for studying the pathogenesis of CNV. Like the results of Lambert *et al*.^9^, our hematoxylin and eosin staining results showed that newly formed vessel tissues, which originated from the choriocapillaris, grew through the break in Bruch’s membrane and ramified between the RPE and the photoreceptor outer segments. The architecture of the inner and outer retina was continuous and unremarkable, which suggested that the CNV mass originated from the choroid, but not the vessels in the retina (Supplemental Fig. S1a). CD31 positive cells (platelet endothelial cell adhesion molecule-1, PECAM-1/CD31, a biomarker of vessel endothelial cells), were observed in the subretinal space, between RPE layer and photoreceptor layer (Supplemental Fig. S1e–h). The CNV appeared on fluorescent fundus angiography (FFA) as a hyperfluorescent lesion with a lace-like margin seven days after laser application. The hyperfluorescence appeared within 1 minute, and increased both in size and intensity at 5 minutes, which indicated leakage from the lesion (Supplemental Fig. S1b,c). Meanwhile, intensive rosette-like hyperfluorescence was also revealed after FITC-dextran angiography on choroidal flat mount 7 days after laser treatment (Supplemental Fig. S1d).

### Spatial and Temporal Expression of Macrophages (MΦ) in CNV Murine Model

**Figure 1:**
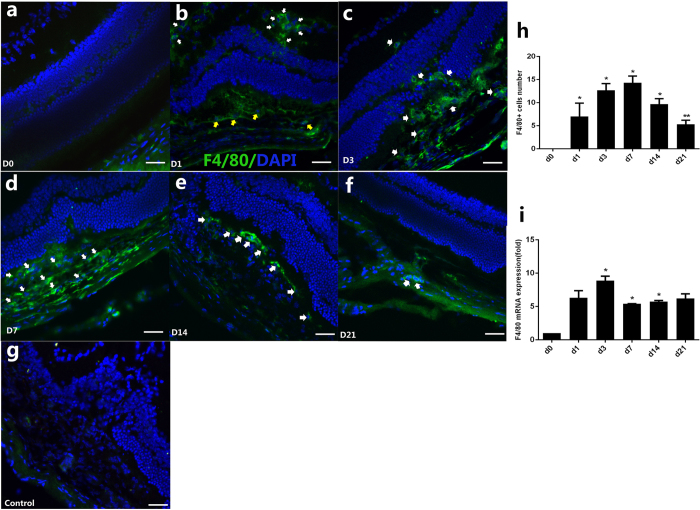
Spatiotemporal expression of macrophage (MΦ) before laser (D0) and on D1, D3, D7, D14, D21 after laser photocoagulation. Immunofluorescence stained images of cross sections showed that the majority of activated macrophages were found in the inner layer of retina (white arrows), meanwhile some macrophages were found in the subretinal space (yellow arrows) on D1 (**b**); cells were found both in outer retina and in subretinal CNV on D3 (**c**), white arrows); the recruitment of F4/80-labeled cells in CNV were significantly prominent on D7 (**d**, white arrows); on D14 and D21, F4/80+ cells decreased significantly (**e,f**), white arrows). Age-matched mouse before laser treatment was shown as D0 (**a**). The negative control was stained with goat anti-mouse IgG antibody Alexa Fluor 488 secondary antibody (**g**). Nuclei were counterstained by DAPI (blue). Scale bar represents 200 um. Bar graphs show mean ± SEM (n = 3) (**h**), *P < 0.05 (Dunnett’s test). Expressions of the F4/80 mRNA in the RPE/choroid complex after laser treatment increased at D1 and peaked at D3 (**i**). Bar graphs show mean ± SEM (n = 12), *P < 0.05 (Dunnett’s test).

Confocal immunofluorescence images of cryosections in the CNV eyes demonstrated that F4/80+ (EGF-like module-containing mucin-like hormone receptor-like 1, EMR1/F4/80, a widely used mouse macrophage antigen marker) macrophages were found as early as day 1 (D1) after the laser treatment (Fig. 1b). Of note, these F4/80+ cells were independent from the laser-induced lesion. They were predominantly located in the inner plexiform layer, inner nuclear layer and outer plexiform layer of the retina, where the retinal vessels are contained. These F4/80 labeled cells were dramatically increased on D3 (Fig. 1c), meanwhile, moving toward to the subretinal space where laser-induced lesion located, from the inner layers of retina. With the growth of the CNV, the F4/80+ cells continuously increased in the lesion and its vicinity at D7 (Fig. 1d), and then decreased at D14 (Fig. 1e) and D21 (Fig. 1f). Normal retinal and choroidal architecture before laser treatment were shown as D0 in (Fig. 1a). The result of F4/80+ cell number counting showed that the cell number increased slowly from D1, peaked at D7, and then significantly decreased at D14 and D21 (*P* < 0.05 at D1, D3, D7, and D14; *P* < 0.01 at D21; Dunnett’s test) (Fig. 1h). Additionally, the quantification of *F4/80* mRNA in the RPE/choroid/sclera complex after laser treatment was consistent with the pattern of cell number, which was increased as early as D1 and was significantly prominent at D3, then decreased gradually after D7 (*P* < 0.05, Fig. 1i).

### Early-but-transient pattern of M1-related markers in the CNV murine model

**Figure 2:**
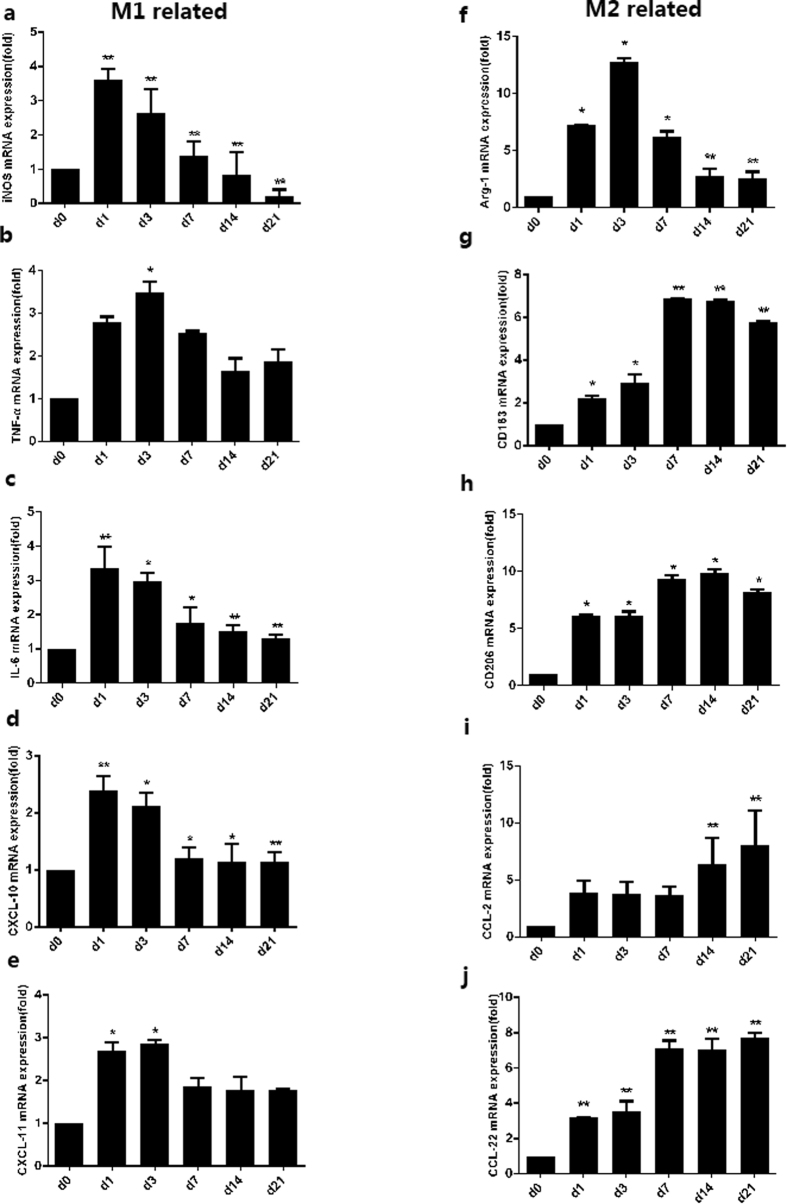
mRNA expressions of M1-related and M2-related markers in mice eyes before laser (D0) and on D1, D3, D7, D14, D21 after laser photocoagulation. M1-related markers included iNos (**a**), Tnf-α (**b**), Il-6 (**c**), Cxcl10 (**d**), Cxcl11 (**e**); M2-related markers included Arg-1 (**f**), Cd163 (**g**), Cd206 (**h**), Ccl2 (**i**), and Ccl22 (**j**). Bar graphs show mean ± SEM (n = 12) **P < 0.01, *P < 0.05 (Dunnett’s test).

**Figure 3:**
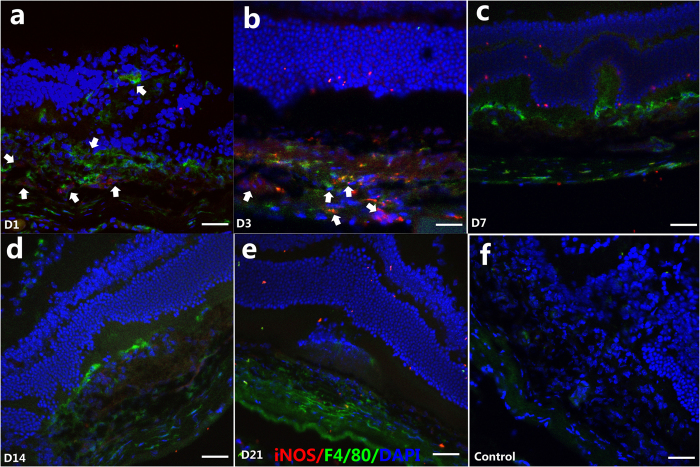
Immunofluorescent double staining of CNV with antibodies F4/80 and iNOS at D1, D3, D7, D14, D21 after laser photocoagulation. Green indicates F4/80; red indicates iNOS and blue indicates DAPI-stained cellular nuclei. Sporadic iNOS-positive cells were found in early stage of laser induced CNV, for example, D1 (**a**, white arrows), and then increased in the CNV lesion on D3 (**b**). At D7 (**c**), D14 (**d**) and D21 (**e**), few iNOS-positive cells were found. The negative control was stained with goat anti-mouse IgG antibody Alexa Fluor 488 and goat anti-rabbit IgG antibody Alexa Fluor 555 secondary antibody (**f**). Scale bar = 200 μm.

Macrophage polarization in the CNV murine model was then investigated in our study. The mRNA expressions of M1-related markers, including *iNOS*, *TNF-α*, *IL-6*, *CXCL10*, and *CXCL11*, in the mouse eyes were measured before (shown as D0) and 1, 3, 7, 14, and 21 days after the laser treatment (Fig. 2a–e, left column). The M1-related mRNA markers were dramatically upregulated on D1 and D3, compared to those on D0 (*P* < 0.05), but were decreased at D7 (*P* < 0.01). Thereafter, the mRNA expression diminished rapidly (e.g. *iNOS*) or decreased slowly (e.g.*, TNF-α*, *IL-6*, *CXCL10*, and *CXCL11*) (Fig. 2a–e). Furthermore, to identify the subtypes of macrophages, the iNOS cells were co-immunostained with F4/80 at different time-points after laser treatment (Fig. 3). The results showed that several iNOS + cells were located within subretinal CNV lesion area on D1, which were co-immunostained with F4/80 (Fig. 3a). However, iNOS was not expressed in the F4/80+ cells located in the inner retina on D1. On D3, iNOS + cells were increased in the CNV locations and their vicinity, and were co-immunostained with F4/80 (Fig. 3b). However, few iNOS + cells were noted at D7, D14, and D21 (Fig. 3c–e). Generally, an early-transient pattern (early elevation and rapid decline) of M1-related markers, both at the mRNA level and the protein level, was observed in the development of CNV.

### Delayed-but-sustained pattern of M2-related markers in the CNV murine model

**Figure 4:**
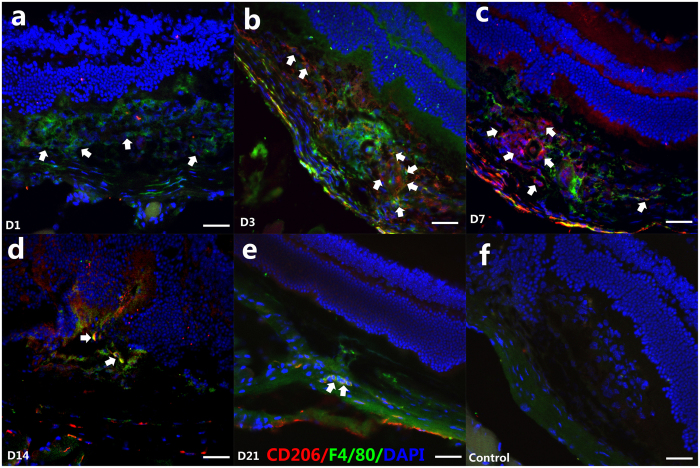
Immunofluorescent F4/80 and CD206 double staining of CNV on D1, D3, D7, D14, and D21 after laser photocoagulation. Green indicates F4/80; red indicates CD206 and blue indicates DAPI-stained cellular nuclei. No CD206-positive cells were detected in CNV at D1 (**a**) (white arrows shown F4/80+ macrophages); F4/80+ /CD206+ macrophages were found at D3 (**b**) (white arrows); at D7, more F4/80+ /CD206+ macrophages were found in the lesion and vicinity (**c**) (white arrows); From D14 to D21, The F4/80+ /CD206 + macrophages were shown on D14 (**d**) and D21 (**e**) (white arrows). The negative control was stained with goat anti-mouse IgG antibody Alexa Fluor 488 and goat anti-rabbit IgG antibody Alexa Fluor 555 secondary antibody (**f**). Scale bar = 200 μm.

The mRNA expressions of M2-related markers, including *Arg-1*, *CD163*, *CD206*, *CCL2*, and *CCL22*, were also measured in this study (Fig. 2f–j, right column). In contrast to the M1 markers, the M2-related mRNA expressions, including those of *CD163*, *CD206*, and *CCL22*, were slightly upregulated on D1 compared to D0 (*P* < 0.05). They gradually increased on D3, significantly peaked at D7, plateaued between D7 to D14, and were sustained to D21 (*P* < 0.05). Moreover, the mRNA expression of *CCL2* was slightly upregulated and plateaued between D1 to D7 (no statistical significance), and increased gradually from D14 to D21(*P* < 0.01). Interestingly, the mRNA expression of *Arg-1* dramatically upregulated on D1, and significantly peaked at D3, then decreased at D7 (*P* < 0.05) and plateaued between D14 to D21 (Fig. 2f–j). In addition, the results of CD206/F4/80 double-staining showed that few CD206 + cells were demonstrated at D1 (Fig. 4a), while a large number of CD206 + cells were detected in the CNV from D3 (Fig. 4b), which overlapped with the F4/80+ cells. Of note, even more CD206 + /F4/80+ cells were found at D7 (Fig. 4c) and D14 (Fig. 4d), before they diminished gradually at D21 (Fig. 4e). These findings together suggest that M2 markers exhibited a delayed but more sustained pattern, if compared to the M1 markers.

### Predominance of M2 over M1 Macrophages in the CNV murine model

After the different mRNA expression and immunofluorescence patterns of M1 and M2 macrophages were identified, we analyzed the ratio of M2 to M1. Two pairs of chemokines were used to represent M2/M1, according to previous studies: 1) the ratio of Arg-1 (an enzyme highly expressed in M2 macrophages) and iNOS (which competes with Arg-1 for a common substrate and is highly expressed in M1 macrophages), and 2) the ratio of CCL22 (a protein secreted by M2) and CXCL10 (a protein secreted by M1). Ratio values of >1 indicated M2 predominance, and ratio values of <1 indicated M1 predominance^10^,^11^ the mRNA ratio at D0 was standardized at 1.

**Figure 5:**
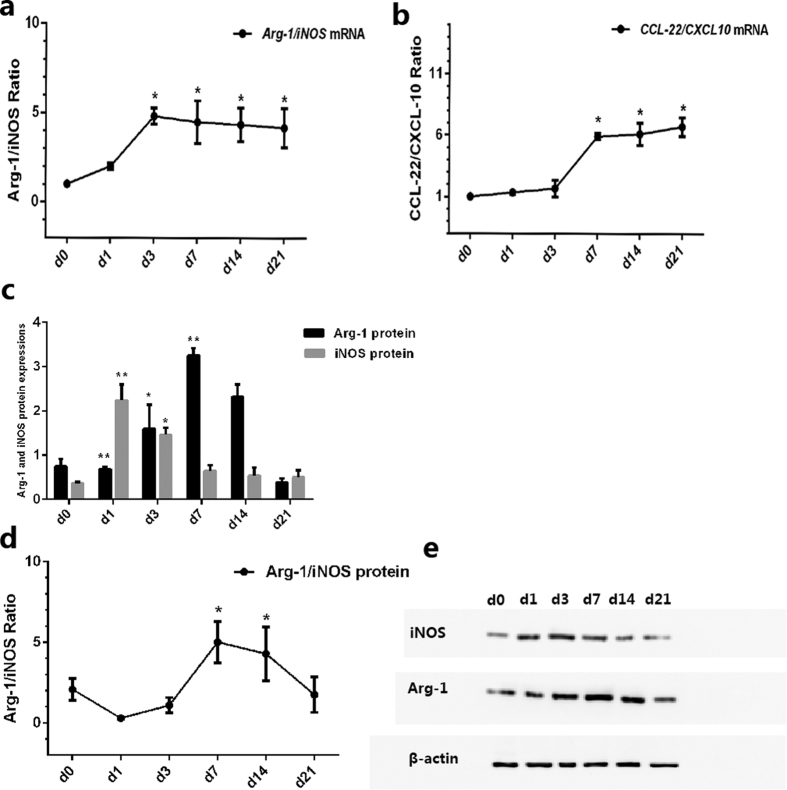
Ratio of M2 to M1 before laser (D0) and on D1, D3, D7, D14, and D21 after laser photocoagulation. (**a**) Relative folds of Arg-1 and iNOS mRNA expression in the RPE/choroid complex after laser treatment. Bar graphs showed mean ± SEM of one representative experiment with 12 samples/time point. (**b**) Ratio of Arg-1 to iNOS. (**c**) Relative folds of CCL22 and CXCL10 mRNA expression in the RPE/choroid complex after laser treatment. Bar graphs show mean ± SEM (n = 12). (**d**) Ratio of CCL22 to CXCL10. (**e**) Relative folds of Arg-1 and iNOS protein by western blot in the RPE/choroid complex. Bar graphs show mean ± SEM (n = 9) (**f**) Ratio of Arg1 to iNOS protein. (**g**) Representative western blot of iNOS and Arg-1protein expressions from laser-induced CNV mice at D0, D1, D3, D7, D14, and D21. β-actin was used as a loading control. **P < 0.01, *P < 0.05 (Dunnett’s test).

The results showed that during the early and late stage of CNV, the increased folding of Arg-1 mRNA is higher than that of iNOS mRNA (Fig. 2a,f), and remarkably higher in CCL22, if compared to that of CXCL10 (Fig. 2d,j). The *Arg-1/iNOS* mRNA ratios were 1.00 (D0), 2.00 ± 0.19 (D1), 4.82 ± 0.46 (D1) (D3), 4.89 ± 1.20 (D7), 4.34 ± 0.94 (D14), and 4.16 ± 1.10 (D21) (*P* < 0.05, Fig. 5a), while the *CCL22/CXCL10* mRNA ratios were 1.00 (D0), 1.33 ± 0.12 (D1), 1.66 ± 0.68 (D3), 5.90 ± 0.24 (D7), 6.10 ± 0.90 (D14), and 6.70 ± 0.76 (D21) (*P* < 0.05, Fig. 5b). There were high M2-associated chemokine (*Arg-1, CCL22*) levels and a high M2/M1 chemokine ratio in the retina/RPE/choroid/sclera complex, compared to tissues from normal control mouse eyes. These results indicated that M2 macrophages, rather than M1 macrophages, likely play a more important role in the development of L-CNV in this model, especially in the late stage.

In addition, we compared iNOS and Arg-1 protein expression levels at different time-points after the laser treatment. The expression of iNOS at D1 (2.24 ± 0.36%, *P* < 0.001) was significantly higher than that at other time-points: 1.46 ± 0.17% at D3, 0.65 ± 0.13% at D7, 0.54 ± 0.18% at D14, and 0.50 ± 0.16% at D21 (Fig. 5c). However, Arg-1 showed low expression at D1 (0.69 ± 0.041%, *P* < 0.001) (Fig. 5c), was increased at D3 (1.60 ± 0.54%, *P* < 0.05), and peaked at D7 (3.26 ± 0.16%, *P* < 0.001). The results for Arg-1/iNOS showed the same pattern, which was 2.08 ± 0.68 (D0), 0.31 ± 0.05 (D1), 1.10 ± 0.48 (D3), 5.02 ± 1.28 (D7), 4.30 ± 1.67 (D14), and 1.76 ± 1.10 (D21) (*P* < 0.05) (Fig. 5d). Taken together, the M2 macrophages comprised a greater proportion of the L-CNV population.

### Predominance of M2 over M1 Macrophages in the Aqueous Humor of Human nAMD Patients

**Figure 6:**
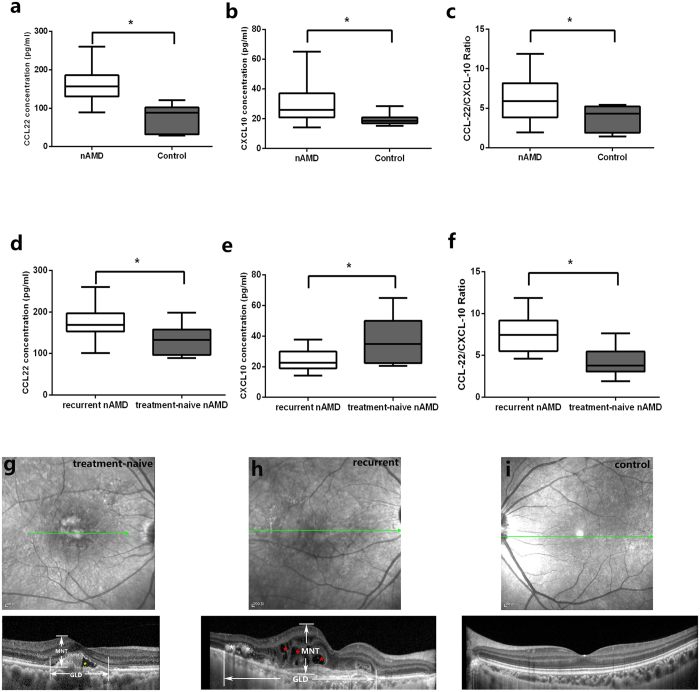
CCL22/CXCL10 in human aqueous humor of nAMD patients and correlation with the OCT features. Box plots showing the minimum and maximum (ends of the whiskers), the median (band near the middle of the box) and interquartile ranges of CCL22 (**a**) and CXCL10 (**b**) in nAMD patients and control. The bottom and top of the box plots represent the 25th and 75th percentile (lower and upper quartiles, respectively); the box plots (horizontal lines by increasing order: minimum, 25%, 50% (median), 75% percentile, maximum) show the distribution of CCL22/CXCL10 ratio for nAMD and control (**c**). The CCL22 was higher in recurrent CNV when compared with that in treatment-naïve patients (**d**), however, CXCL10 was lower in recurrent ones (**e**). CCL22/CXCL10 ratio in recurrent and treatment-naïve nAMD was shown in (**f**). *P < 0.05 (Mann-Whitney U test). (**g–i**) Optical coherence tomography of treatment naïve patient, recurrent patient and control. The horizontal lines with arrows (green) indicated the scan line in SD-OCT. (**g**) Optical coherence tomography of a treatment-naïve patient. The maximal neurosensory thickness (MNT), defined as the maximal vertical distance between inner limiting membrane (ILM) and outer surface of the retinal photoreceptors, and the greatest linear diameter (GLD) of lesion were indicated. Subretinal fluid was noted in this patient (yellow asterisk). (**h**) OCT of a representative patient with recurrent CNV lesion. intraretinal fluid (red asterisk) and hyperreflective foci (white asterisk) were shown. (**i**) A representative OCT in a control individual.

In our recent prior study, sixteen patients with active nAMD who received anti-vascular endothelial growth factor treatment and 12 age-matched controls were enrolled^12^. Among them, seven patients were treatment-naïve and nine were with recurrent CNV. The mean age did not vary significantly between treatment-naïve (70.9 ± 10.0) and recurrent patients (68.8 ± 5.6), with a *P* value of 0.758. The results showed that the median CCL22 was 157.03 pg/ml (25% to 75%:130.51 to 185.91) in the nAMD patients and 88.41 pg/ml (31.92 to 101.97) in controls (*P* = 0.037) (Fig. 6a).CXCL10 was 26.03 pg/ml (20.96 to 37.03) and 18.47 pg/ml (16.95 to 20.90), respectively, with a *P* value 0.004 (Fig. 6b). In the current study, we focused on the ratio of CCL22 to CXCL10, which represents M2/M1. Interestingly, the data showed that CCL22/CXCL10 significantly increased to 5.92 (3.86 to 8.14) in the nAMD group, when compared to that in the control group (4.34, 1.91 to 5.22) (*P* = 0.037) (Fig. 6c).

For the subgroup analysis, CCL22 and CXCL10 were increased both in eyes with recurrent and treatment-naïve nAMD. The levels of CCL22 were increased to 169.38 pg/ml (153.48 to 196.98) in the recurrent group, while is 132.95 pg/ml (96.71 to 157.78) in treatment-naïve eyes (*P* = 0.002) (Fig. 6d). However, the M1 marker, CXCL10, was lower [22.63 pg/ml (18.99 to 30.00)] in the recurrent group, if compared with that in treatment-naïve group [34.88 pg/ml (22.38 to 50.10)] (*P* = 0.012) (Fig. 6e). A further analysis showed that the recurrent group had a significantly elevated CCL22/CXCL10 ratio (7.47, 5.51 to 9.18) when compared to the treatment-naïve group (3.81, 3.06 to 5.45) (*P* = 0.008) (Fig. 6f). The measurements on optical coherence tomography showed that CCL22/CXCL10 was correlated positively with the greatest linear diameter (GLD) of the lesion (ρ = 0.579, *P* = 0.019) and the maximal neurosensory thickness (MNT) (ρ = 0.509, *P* = 0.044), but was not correlated with the presence of subretinal fluid (SRF), subretinal hemorrhage (SRH), hyperreflective foci (HF), or pigment epithelium detachment (PED), with P values of 0.093, 0.562, 0.174, and 0.320, respectively(Fig. 6g–i). Thus, the nAMD patients had higher M2/M1 ratios than the controls, especially the patients with recurrent lesions had the highest ones, indicating that M2 macrophages are predominant and may have a longer and more sustaining effect in the development of CNV.

## Discussion

Although laser-induced CNV is not a true model of nAMD, its condition in animals follows an acute wound-healing tempo rather than the more insidious pathologic changes and low-grade changes to immune responses observed in nAMD, so it is therefore used widely to unravel the mechanisms of CNV^9^,^13^,^14^,^15^,^16^, and also to demonstrate the utility of anti-VEGF therapies^17^,^18^,^19^. In this model, as in humans, macrophage infiltration and macrophage-secreted cytokines and chemokines were found in prior studies^2^,^20^,^21^,^22^,^23^. Caicedo *et al*. found the strong expression of cell adhesion molecules by inner retinal blood vessels, which suggested that the retinal vasculature was activated, facilitating macrophage penetration into the retina through the activated inner blood-retina barrier^22^. In the current study, we found an increase in the density of cells that were immunoreactive for the microglia/macrophage marker F4/80 on D1 and D3, and peaking on D7 (Fig. 1), which was consistent with the development of CNV. This suggests a possible vital role of macrophages in the growth of CNV. Interestingly, in the early stage, for example, at D1 after laser treatment, no typical CNV lesions could be detected (data not shown)^9^,^24^. However, the F4/80+ macrophages were mainly localized in the inner retinal layers, such as the inner and outer plexiform layers, where retinal vessels are located. These macrophages migrated into the photoreceptor layer, arriving at the CNV lesion at D3 and D7.

From the data in our study now, it is difficult to define the origin of these macrophages and to differentiate between the proliferation of resident microglia and the recruitment of hematogenous macrophages. However, Liu *et al*.^25^ investigated the kinetics of macrophage recruitment in laser-induced CNV model via quantitative flow cytometric analysis, and it was demonstrated that accumulating macrophages were migrated from retina and peripheral blood, and were activated at the site of injury prior to exaggerated VEGF expression from RPE. Our results with regard to the presence of macrophages in the early stage after laser treatment, and the spatial dynamic changes from the inner layer toward the CNV lesion via the photoreceptor layer, were consistent with Liu’s results, and provided additional evidences.

Macrophage polarization is highly plastic, depending on the tissue microenvironment, and has been reported in ocular diseases, such as choroidal melanoma and sympathetic ophthalmia^26^,^27^. Macrophages have been reported as both protective and harmful to local tissues in AMD^21^,^22^,^28^. Studies of laser-induced murine CNV have provided insight into the variable and even contradictory effects of macrophages. Cao *et al*. suggested that M2 macrophages are protective in the aging retina and choroid, moreover, they suggested a tendency toward greater M1 activity in atrophy AMD and greater M2 activity in nAMD, which is basically consistent with our data^11^,^29^. In a laser-injury model of nAMD, expression profiling revealed that macrophages that infiltrated into CNV lesions were characterized by expression of M2-type markers, whereas M1-type markers were not induced after the laser injury^30^. To the best of our knowledge, this is the first attempt to demonstrate macrophage polarization in a laser-induced CNV murine model and nAMD patients simultaneously. In our study, we found a mixed profile, with upregulation of both M1 and M2 gene transcripts after laser treatment. We confirmed that macrophages are very flexible in adapting their transcription profile to changes in their microenvironment. In contrast, iNOS, an enzyme highly expressed in M1 macrophages, was dramatically upregulated on the first day after laser treatment, but gradually decreased after D3 and was back to the baseline level on D7. Accordingly, the production of M1 macrophages, with TNF-α serving as a biomarker of M1, was increased on D1, D3, and D7, and only decreased slightly on D14. In addition, the expressions of the inflammatory cytokine IL-6 and of the chemokines CXCL11 and CXCL10, which are produced by activated M1 macrophages, were found to be increased. The quick peaking of these M1 biomarkers would suggest that there is a shift toward M1 shortly after exposure to environmental conditions, which may be the induction of the Th1 response.

On the other hand, our data showed that the balance tilted toward the M2 pathway from D3 after laser treatment. M2-related transcription profiles, including *Arg-1*, were slightly upregulated on D1, then gradually increased, peaking on D7 to D14. Furthermore, cell membrane markers of M2 macrophages, CD163 and CD206, and chemokine CCL22 were noted to be upregulated slightly but continuously from D1, and did not peak until D14. The expression of chemokine CCL22, which is produced by activated M2 macrophages, was found to be increased on D7 and to maintain a relatively high level at least until D21. These data suggest that a shift toward the M2 phenotype occurred after the transient shift to M1, and kept the same tempo in the CNV development. Furthermore, in the current study, CCL22 was found to be significantly increased in the aqueous humor of nAMD patients compared to the controls, and was even higher in patients with recurrent nAMD lesions (Fig. 6). This shift might be due to the role of M2 in anti-inflammatory and revascularization processes. The enhanced presence of anti-inflammatory macrophages in our model offers new opportunities to investigate their role and function in CNV pathogenesis, as well as the immunological signals and inflammatory agents behind their activation and recruitment to the outer retina, a tissue historically thought of as an immunosuppressive environment. Our results are consistent with those of Cao, who reported increased expression of *CCL22* mRNA in human nAMD lesions^11^,^29^. All of these results demonstrate that M1 may be involved in the initial stage of CNV, but that M2 plays a vital role in the middle and advanced stages of CNV development and remodeling.

In order to determine the specific pattern and activation status of these macrophages, we performed intracellular stains for iNOS, which identified M1 macrophages and CD206 production, a hallmark of M2 macrophage differentiation. We observed iNOS + cells within the inner layer of the retina at D1, but CD206 staining was negative, indicating the presence of activated M1 macrophages in the inner layer of retina (Figs 3 and 4). Considered together with the dramatically increased M1 marker genes (*iNOS*, *TNF*-α, *IL-6*, *CXCL11*, and *CXCL10*) on D1, we suggest that M1 macrophages are primarily associated with lesions observed in the outer retina in the early stage, and they may be the main effectors of the inflammatory response. Meanwhile, M2 macrophages are tightly associated with the middle and advanced stages, and are coincidental with the development of CNV. Taken together, M2 macrophages, rather than M1 macrophages, are present in a greater proportion in CNV.

Overall, our work confirms the critical role of macrophages in the development of CNV. We suggest that the dynamic patterns of M1 and M2 in laser-induced CNV are different from each other: an early shift towards M1 immediately, then a delayed-but-sustained shift toward M2. Compared with M1 macrophages, M2 macrophages are predominant and might have a more important effect on the development of CNV. Further investigation is required to understand the mechanisms of macrophage polarization in the pathogenesis of AMD, and how they interact with additional important factors involved in nAMD, such as oxidative injury, complement activation, cell death, and angiogenesis. Targeting these cells and their signaling pathways may be the key to discovering new avenues for the treatment of this disease.

## Materials and Methods

### Mice and Ethics Statement

Pathogen-free C57BL/6 male mice, aged from 6 to 8 weeks old, were used in this study. All procedures and animal care were performed in accordance with the guidelines of the Association for Research in Vision and Ophthalmology on the use of animals in research, and were approved by the Animal Ethical Committee at Zhongshan Ophthalmic Center, Sun Yat-sen University (Permit Number 2013–012).

### Mice Model of CNV

CNV was induced by laser photocoagulation with rupture of Bruch’s membrane as previously described^9^. Briefly, mice at the age of 6 to 8 weeks old were anesthetized with chloral hydrate (100 mg/kg), and pupils were dilated with 1% tropicamide. The photocoagulation process was delivered by Novus Spectra ophthalmic 532 nm diode laser (Lumenis, Inc., Santa Clara, CA), with a cover slide placed on the cornea as a contact lens to view the retina. Burns (50 μm spot size, 0.15 second duration, 150 mW) were then performed 1 or 2 papillary diameter (PD) away from the optic disc. Presence of a vapor bubble, which signified a break in Bruch’s membrane, was required in each laser site during laser application. The success of CNV model was confirmed by fluorescent fundus angiography (FFA) seven days after laser according to the procedure described before^31^,^32^. Briefly, mice were anesthetized, pupils dilated, and 1 mg/kg fluorescein sodium (Alcon Laboratories,Inc.,USA) was injected intraperitoneally. Fluorescent fundus images were taken with the retinal imaging microscope (Micron IV, Phoenix Research Laboratories) at 1, 5 and 10 minutes after injection. The difference of fluorescent intensity between 1 and 5 minute images were recorded as an indicator of CNV vascular leakage. In some mice, the success of CNV model was further confirmed by fluorescent retinal pigment epithelium (RPE)/choroid/sclera flat-mount. Seven days after laser photocoagulation, the mice were perfused with 50 mg/ml fluorescein-labeled dextran (Sigma-Aldrich, St. Louis, MO, USA) from the left ventricular, and euthanized 5 minutes later. Eyes were then enucleated and fixed in 4% formalin for 2 hours. RPE/choroid/sclera complex were carefully dissected, flat-mounted, and examined by fluorescence microscopy. Images were captured with a digital camera (Nikon Instruments Inc., New York, NY).

### Real-Time RT-PCR

To quantitatively analyze the mRNA expressions in eye tissues, real-time RT-PCR assays were performed. Mice at different time-points before and after laser (D0, D1, D3, D7, D14 or D21) were prepared for analysis of the expressions of F4/80 (marker of MΦ), Il-6, Cxcl10, Cxcl11,Tnf-α,iNos (marker of M1) and Ccl2,Ccl22,Cd163,Cd206,Arg-1(marker of M2) and beta-actin (housekeeping gene). Briefly, mice were subjected to laser-induced rupture of Bruch’s membrane with 10 laser spots per eye. Twelve age-matched mice without laser treatment were served as controls. Mice were euthanized and eyes were enucleated. The anterior segments were removed after an annular incision along the equator, and the RNA was isolated from posterior pole RPE/choroid/sclera fractions using Trizol reagent (Invitrogen, Carlsbad, CA, USA) in accordance with the manufacturer’s instructions. Four eye cups from four mice were considered as one sample (12 mice per group). 1 μg of each sample of total RNA, pretreated with DNase I(Promega), was reverse-transcribed into complementary DNA(cDNA) using SuperScript III First-Strand Synthesis System(Invitrogen, Carlsbad, CA, USA). Each PCR was carried out in a 20 uL volume using SYBR Green I Master (Roche) for 10 minutes at 95 °C denaturation, followed by 40 cycles at 95 °C for 30 seconds and 60 °C for 1 minute in Roche480. To determine whether the expression of the potential internal control gene varies under the experimental conditions, the real time PCR data was presented from the replicate cDNAs as 22Ct. The Delta-Delta-Ct (ddCt) method was used for relative quantification. Primers used were listed in Table 1.

### Immunohistochemistry

Immunohistochemistry was performed as previously reported^33^. Eyes were fixed in a mixture of 2.5% glutaraldehyde and 4% paraformaldehyde for 24 hours. For hematoxylin and eosin staining, tissue samples were dehydrated and embedded in paraffin. Serial sections, 8 μm thick, were then cut with a microtome and examined to determine the center of each lesion. For immunofluorescence analysis, the anterior segment and the lens were removed, and the remaining eyecup was cryoprotected with 30% sucrose in 0.1 M PBS (pH, 7.4; 0.15 M NaCl). Eyecups were then embedded in Tissue-Tek OCT compound (Sakura Finetek, Torrance, CA, USA). Sections were cut at 8 μm with a cryostat (Leica Microsystems). Only sections that were cut in the middle of the CNV lesions were included. Sections were incubated with blocking buffer (PBS containing 10% goat serum, 0.5% gelatin, 3% BSA, and 0.2% Tween 20) for 1 hour and were incubated with primary antibodies as listed in Table 2, and were then incubated with fluorescence-conjugated secondary antibody: goat anti-mouse IgG antibody Alexa Fluor 488 and/or goat anti-rabbit IgG antibody Alexa Fluor 555 (1:500 dilution, Cell Signaling Technology, Boston, MA, USA) for 1 h at 37 °C in the dark. Sections were mounted with mounting medium with DAPI (Vectashield Mounting Medium; Vector Laboratories). Pictures were taken under a microscope at 200× (Leica Micro systems) or examined at 400× by laser confocal microscope (LSCM 510 META; Oberkochen, Germany), maintaining identical settings. Blinded cell counting was performed by two separate observers for each confocal images (total area of the visual field imaged at 400× with the CNV lesion in the center).

### Western Blotting

RPE-choroid tissue lysate was used for Western blotting experiments. Freshly dissected posterior eye poles (RPE-choroid tissue after removal of the lens, retina, and anterior segment along the ora serrata) were lysed with lysis buffer containing RIPA and phenylmethylsulfonyl fluoride (PMSF). After centrifugation, the supernatant was used for Western blotting. Protein concentration was determined by the Bradford–Lowry method and the samples were stored at −80 °C until used. Protein samples (20 μg) were loaded onto SDS-PAGE gels and then were transferred to a PVDF membrane (Immobilon-P; Millipore, Bedford, MA, USA). After being blocked with 5% nonfat milk for 1 h, the membrane were incubated with the following primary antibodies at 4 °C overnight: monoclonal rabbit anti-mouse iNOS(1:1000 dilution, Cell Signaling Technology, Boston, MA, USA), polycolonal rabbit anti-mouse Arg-1(1:100 dilution, Thermo Fisher). Equal protein loading was assessed using an monoclonal rabbit anti-β-actin antibody(1:1000 dilution, Cell Signaling Technology, Boston, MA, USA). Protein bands were visualized by incubation with anti-rabbit IgG HRP-linked antibody (1:1000; Cell Signaling Technology, Boston, MA, USA) and chemiluminescence HRP substrates (Immobilon Western; Millipore, Bedford, MA, USA).

### Patients with Active nAMD

Sixteen patients with nAMD who received an intravitreal injection of Ranibizumab (Lucentis; Novartis Pharma AG, Basel, Switzerland) were recruited, and 12 age-matched patients with age-related cataracts who underwent routine phacoemulsification surgery served as controls. The exclusion criteria were: 1) age below 50 years old; 2) history of any other ocular diseases, apart from age-related cataract; 3) any previous intraocular surgery or photodynamic therapy or intravitreal triamcinolone acetonide injection; and 4) previous history of any intravitreal anti-VEGF treatment within the last 6 months in the study eye or within the last 3 months in the fellow eye. All subjects underwent a complete ophthalmic examination, including visual acuity using ETDRS, slit-lamp examination, intraocular pressure measurement, fundus examination, fluorescein angiography, and optical coherence tomography (Heidelberg HRA SPECTRALIS/HRA2, Heidelberg Engineering, Germany). This study was conducted in accordance with Declaration of Helsinki and was approved by the Institutional Review Board of Zhongshan Ophthalmic Center, Sun Yat-sen University. Informed consent was obtained from all patients and controls.

### Analysis of CCL22/CXCL10 in Aqueous Humor of nAMD Patients

Undiluted aqueous humor samples (100–200 μl) were obtained through anterior chamber paracentesis before intravitreal ranibizumab injections in nAMD patients or before cataract surgery in control. In control patients, paracentesis was performed before any conjunctival or intraocular manipulation to avoid breakdown of the blood–aqueous barrier associated with surgical trauma. Samples were snap-frozen and maintained at −80 °C until analysis. To assess the concentration of CCL22 and CXCL10 in aqueous humor, a multiplex ELISA method was used according to the manufacturer’s instructions (Quantibody^®^ Human Chemokine Array, RayBiotech, Inc., Norcross, GA).The concentration and ratio of CCL22/CXCL10 were analyzed with the Quantibody Q-Analyzer (RayBiotech, Inc., Norcross, GA).

### Optical Coherence Tomography Measurement in nAMD Patients

A 6 × 6 mm area of the macular region centered on the fovea was examined by the spectrum domain Optical Coherence Tomography (OCT). All examinations were performed by one well-trained technician, and only images with well-defined retinal layers were selected. All the images were read and analyzed by two independent masked investigators. The presence of intraretinal fluid (IRF), subretinal fluid (SRF), subretinal hemorrhage (SRH), hyperreflective foci (HF) at outer retinal layer, and presence of retinal pigment epithelium detachment (PED) was recorded. Greatest linear diameter (GLD) of lesion, maximal retinal thickness (MRT), defined as the maximal vertical distance between the ILM and the surface of the RPE (including the height of subretinal fluid and subretinal hemorrhages), maximal neurosensory thickness (MNT), defined as the maximal vertical distance between inner limiting membrane (ILM) and outer surface of the retinal photoreceptors were measured as described in prior reports^12^,^34^,^35^,^36^.

### Statistical Analysis

The data were processed and analyzed statistically with SPSS (version 13.0; SPSS, Chicago, IL, USA). For animal study, the means and SEM were calculated for all parameters determined in the study. Data were analyzed statistically using one-way ANOVA. For human study, the data were denoted by median (25^th^ percentile to 75^th^ percentile). Chemokines concentrations were compared using Mann-Whitney U test in different groups. Correlations between cytokine concentrations and OCT data, including GLD of lesion, MRT and MNT, were calculated by Spearman’s correlation test. Two-tailed test or Mann-Whitney U test were used to compare chemokines between nAMD patients with and without IRF, SRF, SRH, HF and PED, respectively. *P* < 0.05 was considered statistically significant.

## Additional Information

**How to cite this article**: Yang, Y. *et al*. Macrophage polarization in experimental and clinical choroidal neovascularization. *Sci. Rep.* **6**, 30933; doi: 10.1038/srep30933 (2016).

## Supplementary Material

Supplementary Information

## Figures and Tables

**Table 1:** Specific Sets of Primers of Real-Time PCR.

Gene names	Primer Sequences
Pan-macrophage related	
* F4/80*	(F)GCATCATGGCATACCTGTTC (R)AGTCTGGGAATGGGAGCTAA
M1-type related	
* Il-6*	(F)CCAAGAACGATAGTCAATTCCAGA (R)CATCAGTCCCAAGAAGGCAAC
* Cxcl-10*	(F)CCAAGTGCTGCCGTCATTTT (R)CTCAACACGTGGGCAGGATA
* Cxcl-11*	(F)GCTGCTCAAGGCTTCCTTATGT (R)ACTTTGTCGCAGCCGTTACT
* Tnf-α*	(F)ACTGAACTTCGGGGTGATCG (R)TGGTTTGTGAGTGTGAGGGTC
* iNos*	(F)CGGCAAACATGACTTCAGGC (R)GCACATCAAAGCGGCCATAG
M2-type related	
* Ccl-2*	(F)TACAAGAGGATCACCAGCAGC (R)ATTCCTTCTTGGGGTCAGCAC
* Ccl-22*	(F)GCTGTGGCAATTCAGACCTC (R)TGACGGATGTAGTCCTGGCA
* Cd163*	(F)ATGCTTCCATCCAGTGCCTC (R)CACAAACCAAGAGTGCCGTG
* Cd206*	(F)GTTCACCTGGAGTGATGGTTCTC (R)AGGACATGCCAGGGTCACCTTT
* Arg-1*	(F)CAGCACTGAGGAAAGCTGGT (R)CAGACCGTGGGTTCTTCACA
Housekeeping gene	
* β-actin*	(F)CATCCGTAAAGACCTCTATGCCAAC (R)ATGGAGCCACCGATCCACA

F, forward primer; R, reverse primer.

**Table 2:** Primary Antibodies Used in This Study.

Antigen	Host	Working Dilution	Manufacturer
F4/80	Rat	1:50	Bio-Rad
iNOS	Rabbit	1:100	Cell Signaling Technology, Boston, MA, USA
CD206	Rabbit	1:100	Abcam, Cambridge, MA, USA
Arg-1	Rabbit	1:100	Thermo
CD31	Rabbit	1:100	Abcam, Cambridge, MA, USA
