# Supplementary material for: Macrophage polarization in experimental and clinical choroidal neovascularization
Source: Sci Rep. 2016 Aug 4;6:30933. doi: 10.1038/srep30933 (PMC4973249; doi:10.1038/srep30933)
Supplement: Supplementary Information [file srep30933-s1.pdf]

Macrophage polarization in experimental and clinical choroidal neovascularization

Yu Yang, M.D., Fang Liu, M.D., Miao Tang, M.D., Miner Yuan, M.D., Andina Hu, M.D.Ph.D., Zongyi Zhan, M.D., Zijing Li, M.D., Jiaqing Li, M.D.Ph.D., Xiaoyan Ding, M.D.Ph.D*， Lin Lu. M.D.Ph.D.*

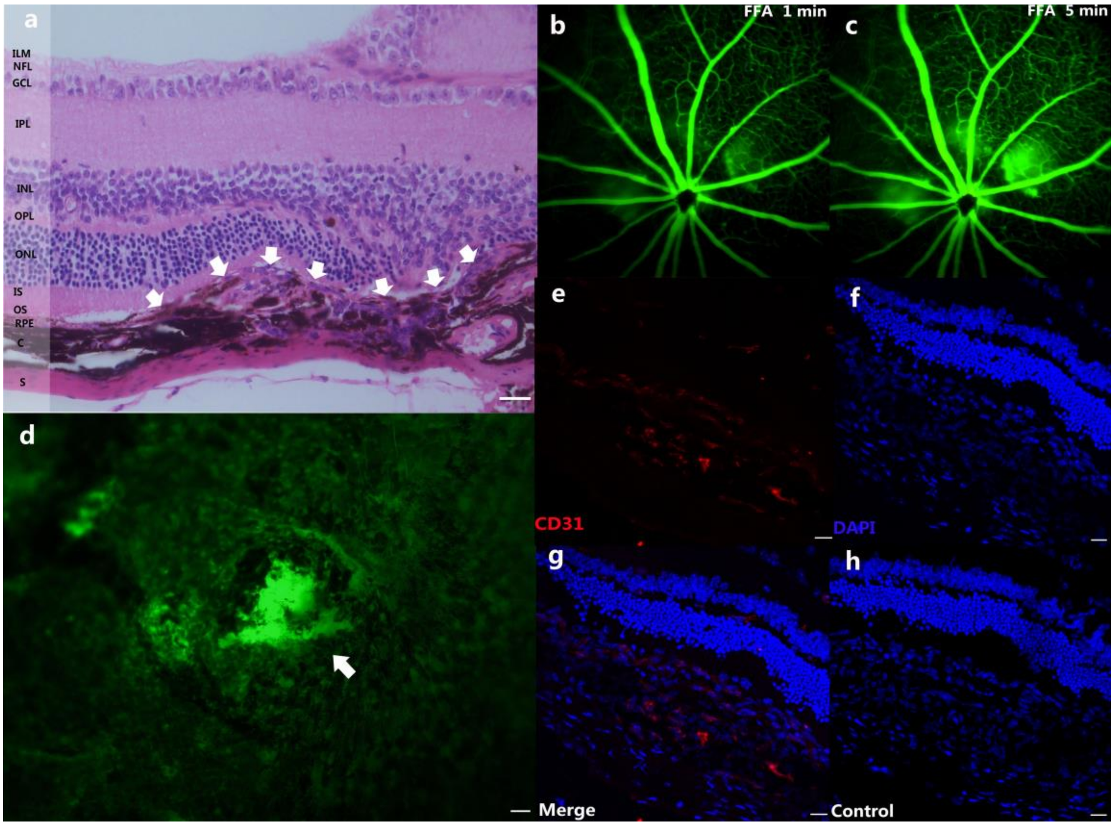

Supplementary Figure S1: Model of murine laser-induced CNV. H&E stained CNV lesion (white arrows) 7 days after laser photocoagulation (D7) (a). CNV leakage was imaged with fundus fluorescent angiography 7 days after laser treatment (b,c). Images were taken at 1(b) and 5(c) minutes after intraperitoneal injection of fluorescein sodium. (d) Representative micrograph of CNV lesions was shown on RPE–choroid flat mounts on D7. CNV was indicated by FITC-dextran angiography (white arrows). Immunostaining of the CNV on D7 with anti-CD31 antibody (e-h). Red indicate CD31 positive (e). Blue indicates DAPI-stained cellular nuclei (f). Merged picture were shown in (g). Negative control was shown in (h). Scale bar = 50 μm. ILM: internal limiting membrane, NFL: nerve fiber layer, GCL: ganglion cell layer, IPL: inner plexiform layer; INL: inner nuclear layer; OPL: outer plexiform layer; ONL: outer nuclear layer; IS: inner segments; OS: outer segments, C: choroid, S: sclera.
